# Increased Sucrose in the Hypocotyls of Radish Sprouts Contributes to Nitrogen Deficiency-Induced Anthocyanin Accumulation

**DOI:** 10.3389/fpls.2016.01976

**Published:** 2016-12-26

**Authors:** Nana Su, Qi Wu, Jin Cui

**Affiliations:** ^1^College of Life Sciences, Nanjing Agricultural UniversityNanjing, China; ^2^School of Land and Food, University of Tasmania, HobartTAS, Australia

**Keywords:** nitrogen deficiency, sucrose, anthocyanins, radish sprouts, soluble sugars

## Abstract

Effects of nitrogen (N) deficiency and sucrose (Suc) addition on regulation of anthocyanin biosynthesis and their relationship were investigated in this study. Radish sprouts subjected to N deficiency had 50% higher anthocyanin accumulation than when grown in Hoagland solution (a nutrient medium with all macronutrients). The contents of endogenous soluble sugars (Suc, fructose, and glucose) in the hypocotyls were also markedly increased by N limitation, with Suc showing the highest increase. Inhibition of carbohydrate biosynthesis by addition of 3-(3,4-dichlorophenyl)-1,1-dimethylurea (DCMU) also eliminated N deficiency-induced anthocyanin accumulation. The latter was further supported by the expression of anthocyanin biosynthesis related genes and decreased activities of nitrate reductase in the presence of Suc. Together our results indicate that N deficiency-induced anthocyanin accumulation was, at least partly, dependent on the increase of the soluble sugar, especially Suc. This work is the first comprehensive study on relationship between N deficiency and sugar content on anthocyanin accumulation in the hypocotyls of radish sprouts.

## Introduction

Anthocyanins represent a large class of flavonoids due to the wide range of chemical structures derived from their synthesis (Andersen et al., 2010). As a natural pigment, anthocyanins provide pigmentation, from red and orange to purple and blue in fruits, seeds and leaves (Zhang and Furusaki, 1999). Besides, anthocyanins are responsible for diverse functions in plants, such as attracting pollinators in petals, aiding seed dispersal (Landi et al., 2015). Anthocyanins can also be important as feeding deterrents, as a producer of photoprotective screens against ultraviolet irradiation damage (Winkel-Shirley, 2001) and as antioxidant molecules protecting against damage by reactive oxygen species (Nagata et al., 2003). These properties have made them to be the focus of research, in which their benefits for human health were also explored. Identified health promoting effects of anthocyanins include stimulating visual acuity and reducing retinal damage (Giampieri et al., 2015), decreasing expression of inflammatory biomarkers (Samadi et al., 2015), diminishing risk of type-2 diabetes mellitus (Guo and Ling, 2015), reducing weight gain (Titta et al., 2010), anti-cancerogenic activity (Forbes-Hernandez et al., 2015) as well as remaining bio-accessible during digestion (Olejnik et al., 2016).

Such diverse and important functions of anthocyanins inspire people to investigate how they are synthesized and by which signaling pathway their synthesis is regulated. By now, it has been shown that anthocyanins are synthesized by phenylpropanoid pathway in which phenylalanine ammonia-lyase (PAL) catalyzes the deamination of phenylalanine to produce precursors (Huang et al., 2010). The subsequent enzymes are chalcone synthase (CHS), chalcone isomerase (CHI), flavanone 3-hydroxylase (F3H), dihydroflavonol4-reductase (DFR), leucoanthocyanidin dioxygenase (LDOX), anthocyanidin synthase (ANS), and UDP-glycose: flavonoid-3-O-glycosyltransferase (UFGT; Passeri et al., 2016). Despite of the distinct biosynthetic pathway of anthocyanins, the regulation of their biosynthesis is very complicated, as the content of anthocyanins in plant tissues could be modulated by various environmental factors, such as UV irradiation, phytohormones, salinity, excessive light, heating, phosphate limitation and diverse biotic stresses (Park et al., 2013; de Aguiar Cipriano et al., 2015; Su et al., 2016).

It has been established that nitrogen deficiency could increase the anthocyanin content in different plant tissues (Lea et al., 2007) by regulating the transcript levels of anthocyanin biosynthesis-related genes (*PAL, CHS, F3H, DFR, LDOX*, and *UFGT*), positive and negative transcription factors (MYBs, small R3-MYB transcription factors; Nemie-Feyissa et al., 2014; Soubeyrand et al., 2014). In addition, high sucrose concentration has also been identified as an efficient environmental factor strongly inducing the anthocyanin accumulation (Nagira and Ozeki, 2004; Ram et al., 2011). In addition to the high expression levels of anthocyanin biosynthesis-related genes (Hara et al., 2004; Solfanelli et al., 2006) sucrose-induction of anthocyanin was related to high osmotic potential in the culture medium (Solfanelli et al., 2006). Besides, results from Loreti et al. (2008) indicate a crosstalk between sucrose and hormones (gibberellins, jasmonate and abscisic acid) in anthocyanin biosynthesis (Loreti et al., 2008).

Although effects of nitrogen and sucrose, together or separate, on regulation of anthocyanin biosynthesis have been largely studied, few of the reports focused on the relationship between sucrose and nitrogen in regulating the pathway of anthocyanin biosynthesis. In this study, we investigate their relationship in regulation of anthocyanin biosynthesis and results showed that increased soluble sugar, especially Suc, contributed to N deficiency-induced anthocyanin accumulation.

## Materials and Methods

### Plant Materials, Growth Conditions, and Treatments

Red skin radish (*Raphanus savitus* L. var. “Cherry Belle”) seeds were soaked in deionized water for about 12 h, and then put in moist gauze to germinate. One-day-old uniform seeds were selected and laid on gauze in plastic containers containing deionized water. Containers were maintained in an incubator (Zhejiang United Saifu Laboratory Instrument Co., Ltd., Ningbo, China) in dark at 25°C for another 48 h. Then the sprouts were treated with different solutions and transferred into another incubator with white light (100 μmol⋅m^-2^⋅s^-1^) for another 24 or 48 h.

### Nutrition Solution Preparation

One liter of Hoagland nutrition solution contains 945 mg Ca(NO_3_)_2_⋅4H_2_O, 506 mg KNO_3_, 80 mg NH_4_NO_3_, 136 mg KH_2_PO_4_, 493 mg MgSO_4_⋅7H_2_O, 13.9 mg FeSO_4_⋅7H_2_O, 18.65 mg EDTA-Na, 2.86 mg H_3_BO_3_, 1.81 mg MnCl_2_⋅4H_2_O, 0.22 mg ZnSO_4_⋅7H_2_O, 0.051 mg CuSO_4_⋅5H_2_O and 0.12 mg Na_2_MoO_4_⋅2H_2_O. For N deficiency, when nutrition solution was prepared, KNO_3_, Ca(NO_3_)_2_4⋅H_2_O and NH_4_NO_3_ were not used, while KCl, CaCl_2_ were added to keep the constant concentration of K and Ca, and others were the same as normal Hoagland nutrient solution. For P deficiency, KH_2_PO_4_ was replaced by KCl, so that P was deficiency in the solution but K was constant. For S deficiency, MgSO_4_ was replaced by MgCl_2_. For K deficiency, KNO_3_ and KH_2_PO_4_ was not used, while more NH_4_NO_3_ and NaH_2_PO_4_ were added to supplement N and P. For Ca deficiency, Ca(NO_3_)_2_4H_2_O was not used, while more NH_4_NO_3_ was add to keep the constant N concentration. For Mg deficiency, MgSO_4_ was not used. After preparation, the pH value of all these solutions was adjusted to 6.0.

### Anthocyanin Analysis

The determination of anthocyanin content in the radish hypocotyls was according to the method developed by Su et al. (2014), which involves measuring the absorbance (530) of extracts.

### Observation of the Hypocotyls Cross Section

Hypocotyls of radish sprouts were transected by a blade and observed under a stereoscopic microscope (Stemi 2000-C; Carl Zeiss, Germany). Pictures were photographed on a color film (Powershot A620, Canon Photo Film, Japan).

### Quantitative and Real-Time RT-PCR Analysis

Total RNA was extracted from radish hypocotyl samples using Trizol extraction reagent (Invitrogen, Gaithersburg, MD, USA) and high purity of RNA with ratio of 260/280 nm > 1.9 was used. First-strand cDNA was synthesized in a 20 μL reaction volume (Thermo Scientific, MD, Lithuania) containing 1 μL of RevertAid M-MuLV reverse transcriptase and 1 μL of oligo (dT)_18_ primer according to the manufacturer’s instructions. A Mastercycler^®^ ep realplex real-time PCR system (ABI7500, MD, USA) with Bestar^®^ SybrGreen qPCR mastermix (DBI, Bioscience Inc., Germany) in a 20 μL reaction volume was used to perform the real-time quantitative PCR reactions according to user manual.

Primer Expressversion 3.0 (Applied Biosystems) was used to design all PCR primers targeting *actin, PAL, CHS, CHI, F3H, DFR, LDOX, ANS*, and *UFGT* (Su et al., 2014). All primers (Supplementary Table 1) were synthesized by Genewiz Bio-engineering Ltd. Company (Suzhou, China). The identification of PAL, F3H, ANS and UFGT in radish genes was based on using their Arabidopsis orthologs for homology search in databank of *R. sativus* available at http://bioinfo.bti.cornell.edu/radish (Shen et al., 2013). The transcription levels were presented as values compared to those of corresponding control samples, after normalization to *actin* expression levels.

### Contents of Soluble Proteins Analysis

Hypocotyl samples (0.05 g FW) were ground in a mortar with liquid nitrogen, and the powder transferred with 3 mL of a phosphate buffered solution (pH 7.0) into centrifuge tubes. After 15 min centrifugation at 13,000 *g* (4°C), 0.1 mL of the supernatant was combined with 5 mL of Coomassie brilliant blue G-250 solution. Two minutes later, the soluble protein content (mg g^-1^ FW) was determined at a wavelength of 595 nm.

### Quantification of Soluble Sugar, Free Amino Acids and Sucrose, Fructose and Glucose

Fresh samples of the radish hypocotyls (1 g) were ground in a mortar with 2.5 mL of distilled water. The homogenates were centrifuged at 10,000 rpm for 15 min, and the supernatant was used to analyze the contents of soluble sugar, free amino acid, glucose (Glu), fructose (Fru) and sucrose (Suc). The soluble sugar content was determined using the sulfuric acid anthrone method with measurements conducted on a spectrophotometer (UV-5200 spectrophotometer, Shanghai Metash Instruments Co., Ltd, Shanghai, China) at a wavelength of 630 nm (Morris, 1948). Free amino acids content was determined using the ninhydrin method with measurements done at a wavelength of 570 nm (Moore and Stein, 1948).

Glc, Fru, and Suc levels were determined by the method from Gordon et al. (1997). Briefly, for the determination of Glc, the extract was incubated with 200 μL of buffer (50 mM imidazole, 1 mM MgCl_2_, 0.75 mM NAD, 0.85 mM ATP) containing 0.04 unit of Glc-6-P dehydrogenase and 0.1 unit of hexokinase, and then 50 μL samples were assayed in 96-well plate. For Fru and Suc, phosphoglucose isomerase (0.4 unit/well) and acid invertase (20 units/well), respectively, were added in the mix before measurement using a plate reader (TECAN Infinite M 200) at 340 nm.

### Analysis of the Activities of Sucrose Synthase (SS), Sucrose Phosphate Synthase (SPS), Glutamine Synthase (GS) and Nitrate Reductase (NR)

Hypocotyl samples (0.05 g FW) were ground in a mortar with liquid nitrogen and then the powder was transferred together with 3 mL of a phosphate buffered solution (pH 7.0) into centrifuge tubes. After 15 min centrifugation at 13,000 *g* (4°C), the supernatant was collected for the analysis of enzymes activities. Enzyme activities are expressed as moles of metabolite generated/consumed per milligram of protein per unit of time.

SPS activity measurements were based on the method from Klann et al. (1993), and 1 U = 0.5 μmol h^-1^.

SS activity was determined according to Klann et al. (1993). Absorbance was measured at 540 nm using Fru as a standard with 1 U equal to 0.5 μmol h^-1^.

For NR, radish hypocotyl tissue (0.1 g) was ground in 1 mL of buffer containing 50 mM KH_2_PO_4_-KOH buffer, pH 7.5, 2 mM EDTA, 2 mM dithiothreitol, and 1% polyvinylpolypyrrolidone. NR activity was measured according to the method from Reguera et al. (2013). The activity of NR was expressed as the amount of NO_2_^-^ produced per unit of fresh weight per hour and 1 U was 0.5 μmol h^-1^.

GS were determined according to O’Neal and Joy (1973), 1 U was 1 μmol h^-1^.

### Statistical Analysis

Values presented are means ± standard deviation (SD) of three replicates. Data was subjected to analysis of variance (ANOVA), and mean values were compared by Duncan’s and Tukey’s multiple range test (*p* < 0.05). All the statistical analyses were performed using SPSS 19.0 for Windows.

## Results

### Radish Sprouts Grown in Nutrient Solutions Show Lower Anthocyanin Content

**FIGURE 1:**
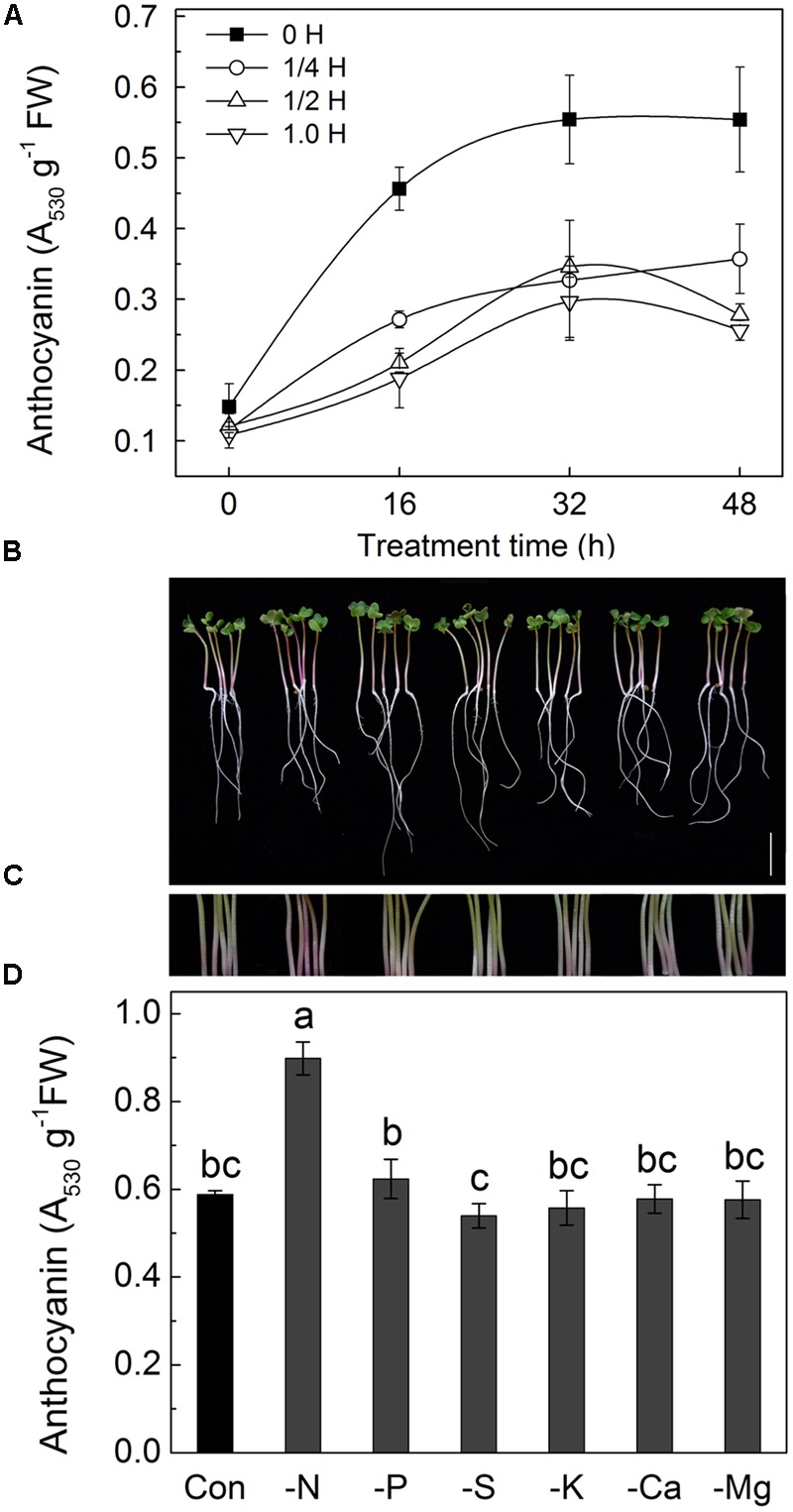
**Changes of morphology, hypocotyl color and anthocyanin content in hypocotyls of radish sprouts grown in different nutrient solutions. (A)** Anthocyanin contents in hypocotyls of radish sprouts grown in different Hoagland nutrient solutions. The germinated radish seeds first were grown in deionized water under dark condition for 48 h, and then the deionized water was changed for different nutrient solutions: deionized water (0 H), ¼ strength Hoagland solution (1/4 H), half-strength Hoagland solution (1/2 H) and full-strength Hoagland solution (1.0 H). After that, the radish sprouts were transferred into incubators with light intensity of 100 μmol⋅m^-2^⋅s^-1^, and this time point was considered as 0 h time point. The hypocotyls were collected after 0, 16, 32, and 48 h of growth for anthocyanin analysis. **(B–D)**, changes of morphology **(B)**, hypocotyl color **(C)** and anthocyanin content **(D)** in hypocotyls of radish sprouts grown in different nutrient solutions with various elemental deficiencies. After 48 h growth in dark, the deionized water was changed for different nutrient solutions with element deficiency, and sprouts were transferred into light for another 48 h. Modified Hoagland solutions were deficient in a single nutrient to accomplish nitrogen (-N), phosphorus (-P), sulfur (-S), potassium (-K), calcium (-Ca) and magnesium (-Mg) deficiencies. The bar = 2 cm in **(B)**. Data are means ± SD (*n* = 3). Mean values were compared by Duncan’s and Tukey’s multiple range test (*p* < 0.05). Data labeled with different lower case letters are significantly different.

In our preliminary experiments, an interesting phenomenon was observed in which the color of radish hypocotyls varied depending on the composition of the culture solution used. As the main pigment responsible for the red color in radish sprouts are anthocyanins, the anthocyanin contents in the hypocotyls of radish sprouts grown in Hoagland solutions of different strengths were measured (**Figure 1A**). The results showed that anthocyanin contents increased with duration of the treatment, peaking at 32 h, after which anthocyanin contents remained at relative steady levels. In addition, amount of anthocyanins decreased with increase of the strength of Hoagland solution, with almost twofold higher level being measured in radish sprouts grown in deionized water compared with those grown in full strength Hoagland solution.

### Radish Sprouts Accumulate More Anthocyanins in Nitrogen Deficient Conditions

The above finding was used to further investigate whether a specific component of the Hoagland solution effected the anthocyanin level. A single-factorial experiments were designed with one of the nutrients [nitrogen (N), phosphorus (P), sulfur (S), potassium (K), calcium (Ca) and magnesium (Mg)] being removed at a time from the Hogland solution. Radish seedlings were grown in each of the modified Hogland solutions and assessed for anthocyanin contents (**Figures 1B–D**). As shown in **Figure 1B**, the morphological characteristics of radish sprouts grown in various conditions were similar, while a considerable increase of anthocyanin content was observed only in the absence of N in the nutrient solution (**Figures 1C,D**), suggesting that N element is one of the major contributing factors to the observed phenomenon.

### Nitrogen Addition Decreases Anthocyanin Accumulation and Soluble Sugar Content

**FIGURE 2:**
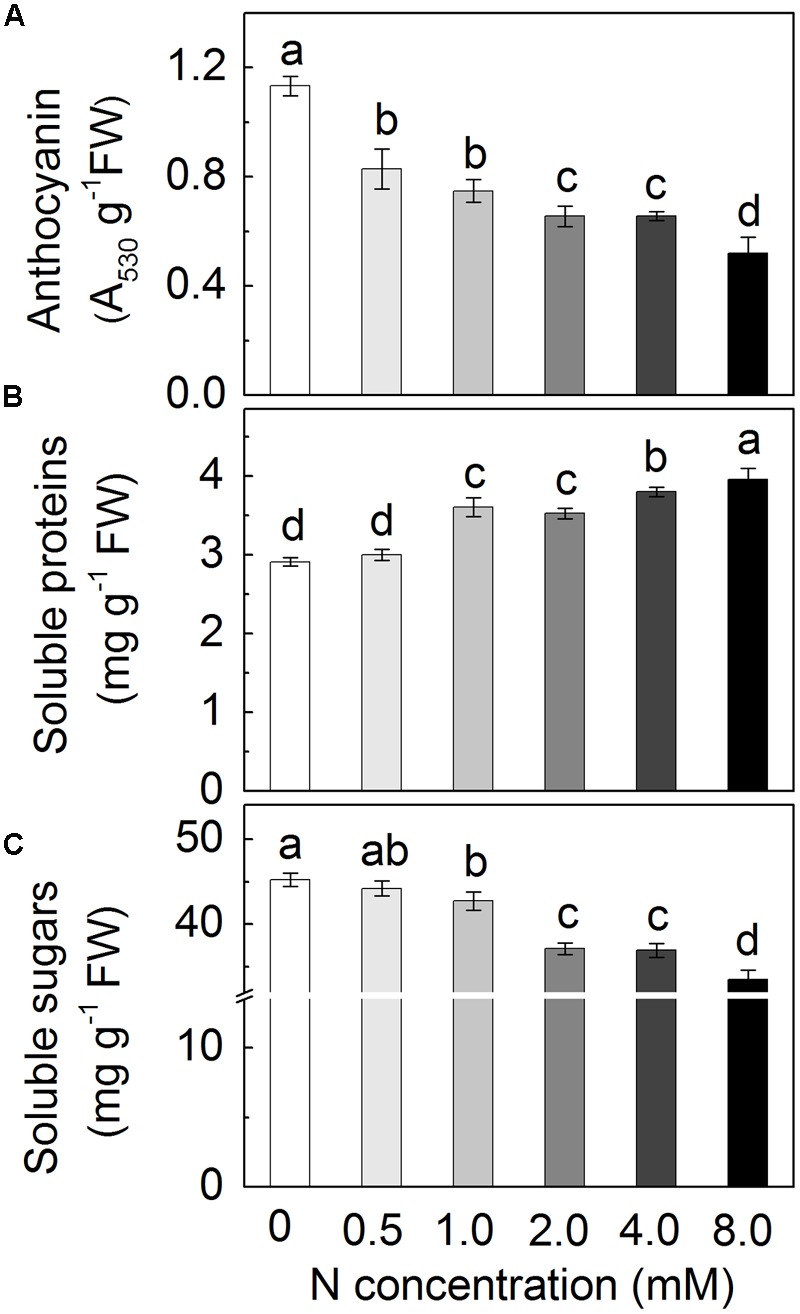
**The anthocyanin content **(A)**, soluble proteins **(B)** and soluble sugars **(C)** in the hypocotyls of radish sprouts under ascending N concentrations from 0 to 8.0 mM.** Data are means ± SD (*n* = 3). Data labeled with different lower case letters are significantly different at *P* < 0.05.

To investigate the effects of N in the regulation of anthocyanin biosynthesis, radish seedlings were grown in Hoagland solutions containing various amounts of N (from 0 to 8.0 mM) and anthocyanin contents were assessed in the hypocotyl tissues (**Figure 2A**). As expected, the contents of anthocyanin accumulated depended on the N concentration in the nutrient solution with a progressive decrease of the anthocyanin levels according to the increase of N concentration. Additionally, hypocotyls of sprouts grown under excessive N showed more soluble proteins but less soluble sugars (**Figures 2B,C**).

### N Deficiency Increases the Contents of Soluble Sugar and Exogenous Addition of Sugars Enhance Anthocyanin Accumulation

**FIGURE 3:**
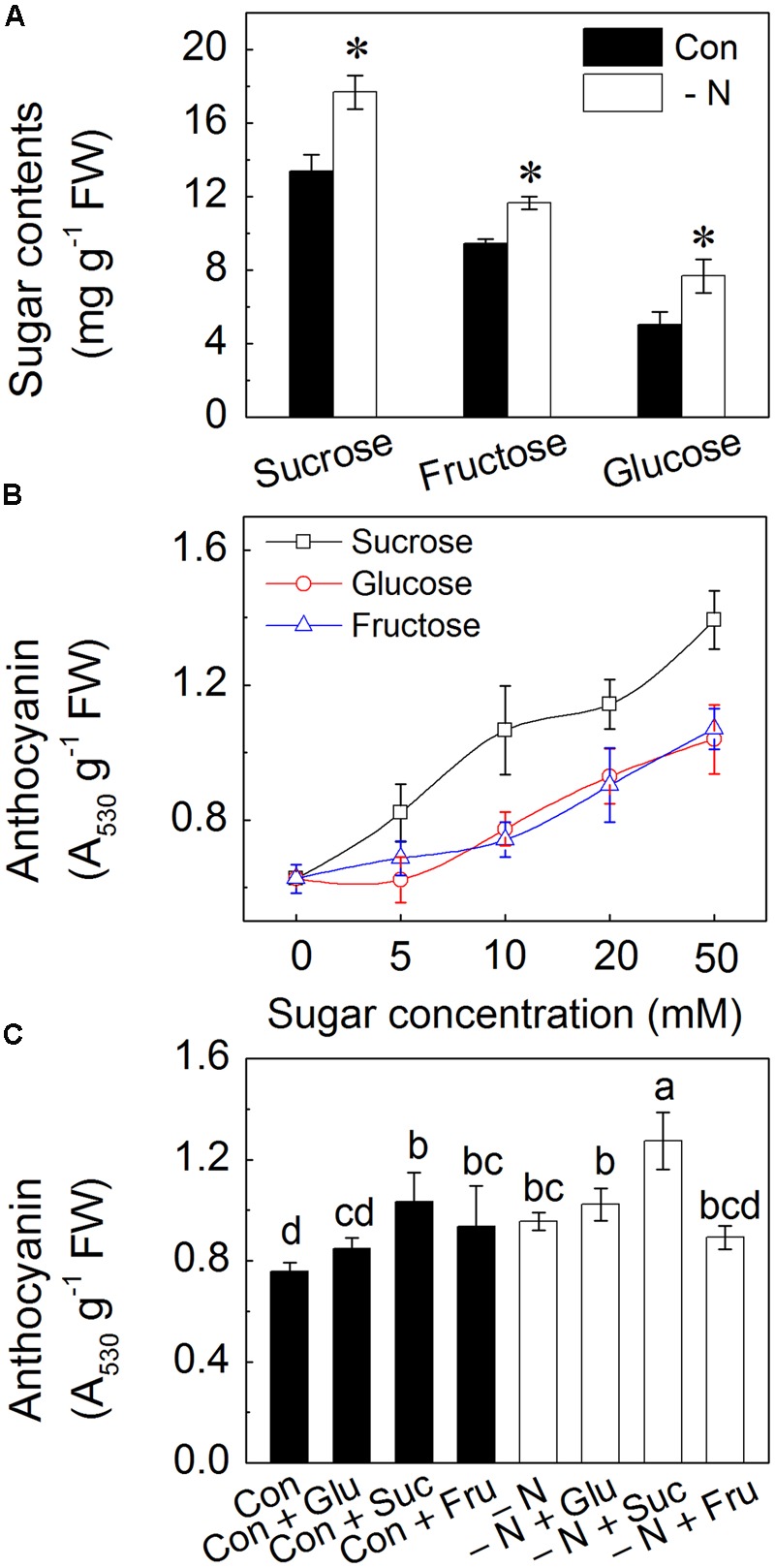
**Effects of N deficiency and sugar supplementation to the growth solution on the sugar content and anthocyanin accumulation in the hypocotyls of radish sprouts. (A)** Amounts of sucrose, fructose, and glucose contents accumulated in the presence (dark) and the absence (white symbols) of N in the growth medium. Control (con) represents growth in a full strength Hoagland solution while N deficiency is indicated as “-N.” The asterisk represents the significance at *P* < 0.05 between respective pairs (i.e., presence vs. absence of N). **(B)** Effects of ascending concentrations of sucrose, glucose, and fructose on anthocyanin accumulation in the normal Hoagland solution. Seedlings were grown at various sugar concentrations (0, 5, 10, 20, and 50 mM) in Hoagland solution. **(C)** Effects of the N deficiency and different sugars (Glu, Suc and Fru) on the anthocyanin contents in the hypocotyls of radish sprouts. After 48 h growth in dark, the deionized water was changed into different nutrient solutions, and sprouts were transferred into light for another 48 h when the samples were collected for analysis. Data are means ± SD (*n* = 3). Data labeled with different lower case letters are significant differences at *P* < 0.05.

Suc, Fru and Glu are three main forms of soluble sugars in plants (Rosa et al., 2009). In the hypocotyls of radish sprouts, the concentration of Suc was the highest (approximate 13 mg/g FW), followed by Fru (approximate 10 mg/g FW), with relatively low concentration in Glu (approximate 5 mg/g FW) detected (**Figure 3A**). When subjected to N deficiency, levels of all these soluble sugars were considerably increased, with contents being 40, 20, and 40% higher for Suc, Fru and Glu, respectively (**Figure 3A**). Addition of these soluble sugars enhanced accumulation of anthocyanins in the hypocotyls. The biggest changes were observed on addition of Suc with over twofold increase under 10 mM Suc concentration. The response was dose-dependent, with increase in anthocyanin contents in response to increase of sugar concentrations (**Figure 3B**). Exogenous addition of 50 mM soluble sugars inhibited the growth of radish sprouts, thus a concentration of 10 mM was selected for the following experiments.

To further determine the relationship between N deficiency and soluble sugars in anthocyanin accumulation, sprouts were grown in N-deficient conditions with addition of different soluble sugars. Addition of 10 mM Suc significantly increased the anthocyanin accumulation in the hypocotyls compared to control, whereas only slight enhancement of anthocyanin content was observed under addition of 10 mM Glu and Fru (**Figure 3C**). Therefore, effect of Suc on the anthocyanin biosynthesis was explored further. In addition, we observed that sprouts subjected to addition of Glu or Suc under N deficiency showed much higher level of anthocyanins as those grown under Glu or Suc with presence of N (**Figure 3C**).

### N Deficiency-Induced Anthocyanin Accumulation Disappears When the Biosynthesis of Carbohydrates Is Inhibited

**FIGURE 4:**
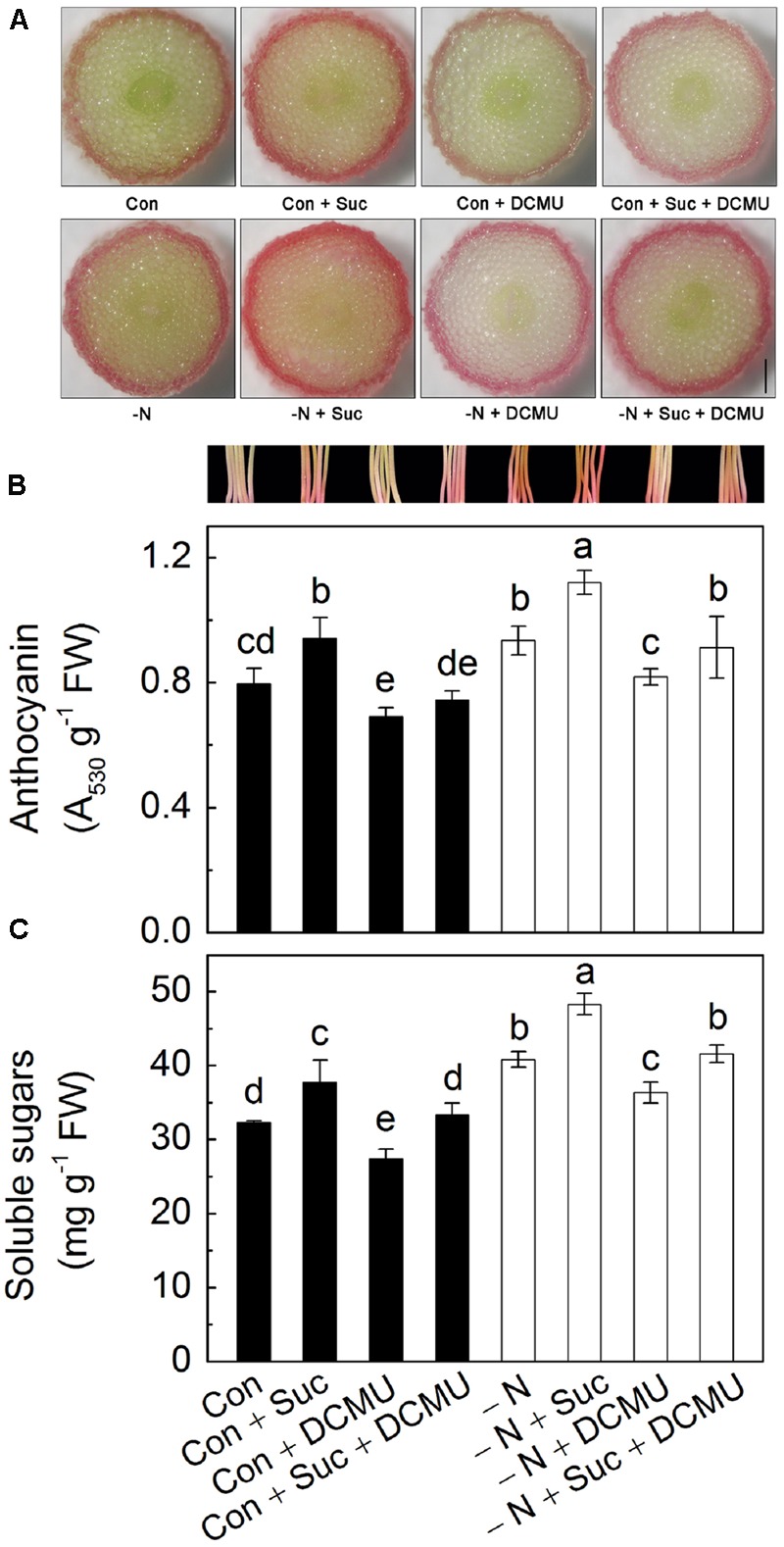
**The transection of hypocotyls **(A)**, hypocotyl color and anthocyanin content **(B)** and amount of soluble sugars **(C)** accumulated in the hypocotyls of radish sprouts under different growth conditions.** Different nutrient solutions used for seedlings growth were modified Hoagland solutions containing N (control, Con, dark) or deficient in N (-N, white symbols) and various modifications of the two, specifically: Hoagland full strength solution containing 10 mM sucrose (Con + Suc), 10 μM DCMU (Con + DCMU), 10 mM sucrose and10 μM DCMU (Con + Suc + DCMU); N-deficient Hoagland solution containing 10 mM sucrose (-N + Suc), 10 μM DCMU (-N + DCMU), 10 mM sucrose and10 μM DCMU (-N + Suc + DCMU). Bar in A is 0.3 mm. Data are means ± SD (*n* = 3). Data labeled with different lower case letters have significant differences at *P* < 0.05.

The results of cross section showed that anthocyanins mainly accumulated in the epidermis of the hypocotyls of radish sprouts (**Figure 4A**), and compared with control, addition of Suc markedly increased the content of anthocyanins in the hypocotyls. DCMU [3-(3,4-dichlorophenyl)-1,1-dimethylurea] inhibits photosynthetic electron transport, and consequently reduce the production of chemical energy (ATP) and reducing power (NADPH), resulting in inhibition of carbon fixation process and, eventually, sugar biosynthesis (Jeong et al., 2010). Addition of DCMU decreased the anthocyanin accumulation significantly, which was reverted by Suc supplementation (**Figures 4A,B**). N deficiency also induced observable increase in anthocyanin content compared to control. Addition of 10 mM Suc to N-deficient solution enhanced anthocyanin content by 40%, whereas addition of DCMU completely eliminated the effect (**Figure 4B**). Supplementing Suc and DCMU together to growth solution negated each other leading to anthocyanin content being similar to those in N-deficient solution. Change of growth conditions also affected amounts of soluble sugars accumulated in the hypocotyls of radish sprouts, with addition of Suc causing their substantial increase under both growth conditions (the presence and absence of N in the nutrient solutions, **Figure 4C**). The trend observed for soluble sugars was similar to changes in anthocyanin levels described above (**Figures 4B,C**, respectively).

### Expressions of Anthocyanin Biosynthesis-Related Genes Are Up-Regulated by Suc and N Deficiency and Down-Regulated by DCMU

**FIGURE 5:**
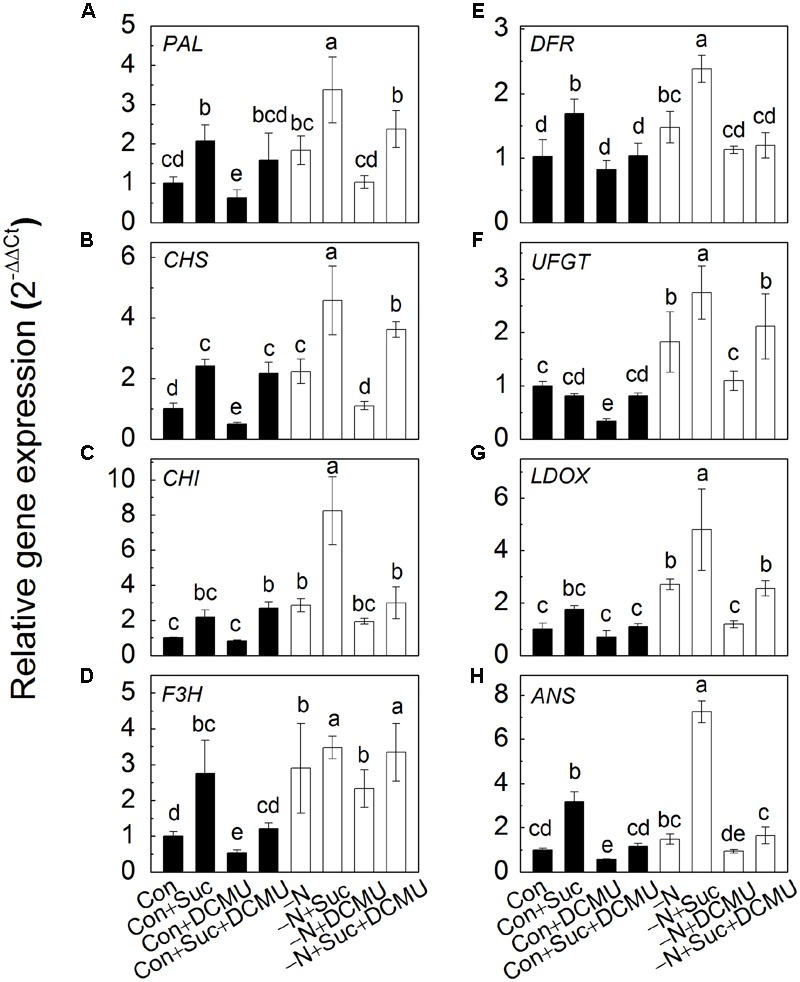
**The expression of anthocyanin biosynthesis-related genes (*PAL*, **A**; *CHS*, **B**; *CHI*, **C**; *F3H*, **D**; *DFR*, **E**; *UFGT*, **F**; *LDOX*, **G**; *ANS*, **H**) in the hypocotyls of radish sprouts grown in different solutions.** After 48 h in dark, the deionized water was changed for different nutrient solutions such as full strength Hoagland solution (control, Con, dark symbols) and its variations: with addition to Hoagland solution of 10 mM sucrose (Con + Suc), 10 μM DCMU (Con + DCMU), 10 mM sucrose and10 μM DCMU (Con + Suc + DCMU); N deficient Hoagland solution (-N, white symbols) and its variations: with addition to Hoagland solution of 10 mM sucrose (-N + Suc), 10 μM DCMU (-N + DCMU), 10 mM sucrose and 10 μM DCMU (-N + Suc + DCMU). Sprouts were transferred into light for another 48 h and the samples were collected for analysis. Data are means ± SD (*n* = 3). Data labeled with different lower case letters have significant differences at *P* < 0.05.

In addition to the content of anthocyanins, the transcript levels of anthocyanin biosynthesis-related genes (*PAL, CHS, CHI, F3H, DFR, UFGT, LDOX*, and *ANS*) were measured under different treatments. As shown in **Figure 5**, the trends of changes in gene transcriptions were in agreement with the changes of anthocyanin content under the same growth conditions. Indeed, sprouts had substantially higher expression of anthocyanin biosynthesis-related genes under N deficiency compared to genes expressed under control conditions (full strength Hoagland solution) either in the presence or absence of Suc, DCMU and their combination. The highest transcript levels of the genes were observed in the hypocotyls of sprouts subjected to N deficiency with addition of Suc. The latter phenomenon, however, disappeared when sprouts were treated with DCMU.

### N Deficiency Induces Activity of Sucrose Synthase (SS), while Suc Addition Induces a Decrease in the Activity of Glutamine Synthase (GS) and Nitrate Reductase (NR)

**FIGURE 6:**
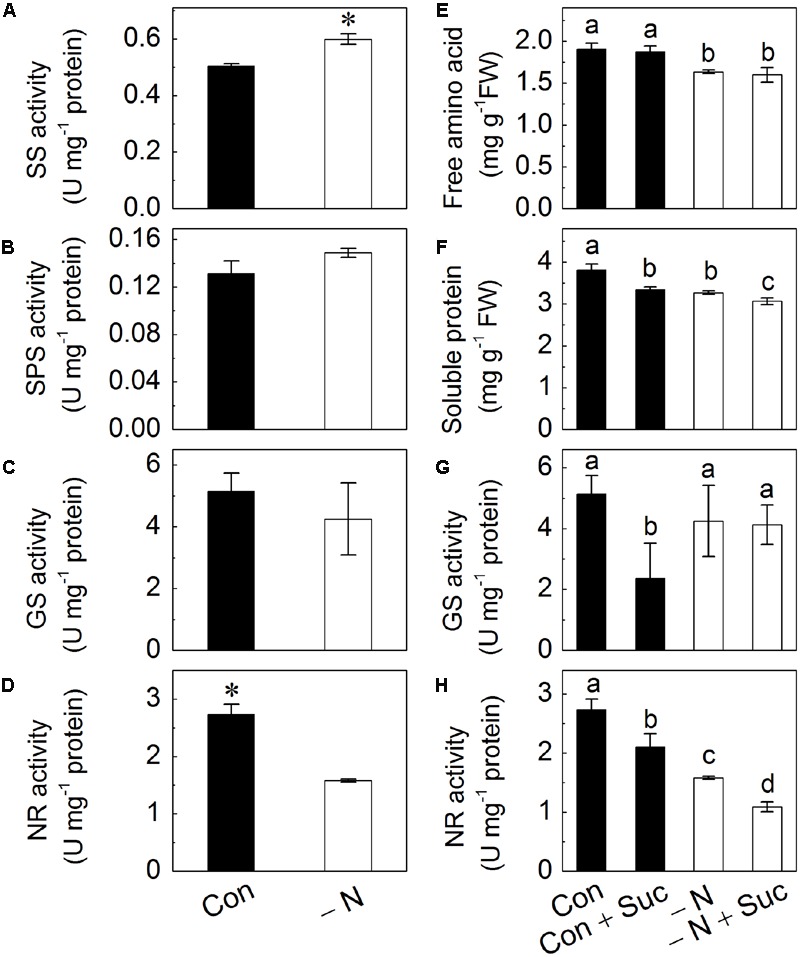
**Effects of N deficiency on the activity of sucrose synthase (SS; **A)**, sucrose phosphate synthase (SPS; **B**), glutamate synthase (GS; **C**) and nitrate reductase (NR; **D**). Effects of the presence of N and sucrose in the growth solution on amounts of free amino acids **(E)**, soluble proteins **(F)** and activities of GS **(G)** and NR **(H)** in radish sprouts.** After 48 h growth in dark, the deionized water was changed for different nutrient solutions, which were full strength Hoagland solution (control, Con), with addition of 10 mM sucrose (Con + Suc), N-deficient Hoagland solution (-N), with addition of 10 mM sucrose (-N + Suc). After change of solution, sprouts were transferred to light for another 48 h, and the samples were collected for analysis. Data are means ± SD (*n* = 3). Data labeled with different lower case letters have significant differences at *P* < 0.05.

The activity of SS, sucrose phosphate synthase (SPS), GS and NR were determined to further investigate the effects of N deficiency on Suc biosynthesis and N metabolism (**Figures 6A–D**). Results showed that N deficiency positively regulated SS activity (**Figure 6A**) and negatively regulated NR activity with the latter being reduced by nearly twofold (**Figure 6D**). No effects on the activities of SPS and GS were found (**Figure 6B,C**). Nitrogen deficiency also negatively affected free amino acids, soluble proteins levels, and NR activity but not activity of GS (**Figures 6E–H**).

We also assessed effects of Suc presence in the growth solutions on levels of amino acids and soluble proteins and enzymes activities. Addition of Suc to full strength Hoagland solution (control) significantly reduced the content of soluble proteins and activities of GS and NR (**Figures 6F–H**). Suc did not affect levels of free amino acids in either control or N-deficient solution (**Figure 6E**). No change was also found in GS activity under N-deficient condition (**Figure 6F**). At the same time addition of Suc to N deficient solution led to a decrease of NR activity and to amounts of soluble proteins, similar to the trends observed under control conditions (**Figures 6F,H**).

## Discussion

With more attention being focused on nutritious and healthy food, radish sprouts have found their way to human diet due to their high levels in antioxidants, carotenoids, vitamin C, fiber, flavonoids and glucosinolates (Takaya et al., 2003; Marton et al., 2010). Red skin radish sprouts have higher nutrition value due to the red hypocotyls which are rich in anthocyanins (Papetti et al., 2014).

In the present study, cultivating radish sprouts in deionized water led to substantially more anthocyanins in the hypocotyls than those in nutrient solutions (**Figure 1A**). Further assay showed that this phenomenon was due to N deficiency, which was supported by (1) removing other ions from the Hoagland nutrient solution (such as P, S, K, Ca, and Mg) had no effect on anthocyanin accumulation; (2) more anthocyanins accumulated when sprouts were grown in N deficient solution (**Figures 1C,D**). These results are in agreement with earlier reports (Nemie-Feyissa et al., 2014; Soubeyrand et al., 2014; Medina-Perez et al., 2015) indicating that N deficiency affects regulation of anthocyanin biosynthesis. Other authors reported enhancement of anthocyanin content by P deficiency in strawberry fruits, flower stalk of Chinese kale and Arabidopsis leaves (Valentinuzzi et al., 2015; Khan et al., 2016), however, that was not the case in our study. Similar to our findings, Jia et al. (2015) reported that there was no effect of P deficiency on the anthocyanin content in tobacco leaves. Additionally, it was suggested that macronutrients deficiency might cause an abiotic stress in plants, and abiotic stress would induce the anthocyanin enhancement (Van den Ende and El-Esawe, 2013). Absence of increase in anthocyanin content in radish sprouts observed in our experiments under P, S, K, Ca or Mg deficiency might be explained by early developmental stages of the sprout used in our experimental conditions (3-day old) when abiotic stress induced by elemental deficiency has not been appeared yet. Compared results in **Figure 1A** with those in **Figure 1D**, the increase of anthocyanins induced by deionized water was more than N deficiency, which might result from the abiotic stresses from starvation, hypo-osmotic and non-optimal pH conditions in deionized water.

To further confirm the negative effects of nitrogen on anthocyanin accumulation, radish sprouts were grown under different N concentrations. As expected, increase of N concentration in the growth solutions led to a decrease anthocyanin content (**Figure 2A**). On the contrary, soluble proteins increased according to the ascending N concentration (**Figure 2B**). That was reasonable, as N is a crucial component of proteins. An interesting result was that contents of soluble sugars showed a similar descending trend with anthocyanin accumulation (**Figure 2C**) indicating that there may be a negative effect of N on the content of soluble sugars. This hypothesis was confirmed by results in **Figure 3A**, in which the contents of Suc, Glu and Fru, that comprise main soluble sugars (Rosa et al., 2009), were all significantly increased in sprouts grown under N deficiency. This phenomenon has been reported by a number of researches showing that high nitrogen application resulted in reduction of the sugar content (Bénard et al., 2009; Prvulović et al., 2009). The increased sugar contents positively affected anthocyanin content in hypocotyls of sprouts. This was shown to occur in the presence of Suc, Glu and Fru, with Suc being most effective (**Figure 3B**). Similarly a number of researches reported the positive effect of soluble sugars on anthocyanin accumulation (Nagira and Ozeki, 2004; Loreti et al., 2008; Ram et al., 2011). A study from Solfanelli et al. (2006) indicated that Suc is specific in the sugar-dependent up-regulation of the anthocyanin synthesis pathway. While both Suc addition and N deficiency led to increase in anthocyanin level, the relationship between the two factors remains largely unexplored. In this work we investigated causative relationship between N levels and amounts of soluble sugars in the regulation of anthocyanin biosynthesis and involvement of specific enzymatic pathways.

To investigate the relationship between N and Suc in modulating of anthocyanin accumulation, radish sprouts were treated with exogenous soluble sugars under normal or N deficiency condition. Addition of Suc dramatically increased the anthocyanin content in hypocotyls and this increase was further enhanced under N deficiency condition (**Figure 3C**), implying a potential role of Suc in N deficiency-induced anthocyanin accumulation. To validate this hypothesis, an inhibitor of carbohydrate biosynthesis, DCMU, was used. Addition of DCMU to radish sprouts markedly reduced the content of soluble sugars and anthocyanins in hypocotyls, and this inhibition was reversed by application of Suc to the growth solution (**Figure 4**), indicating involvement of sugar biosynthesis in the observed changes. Additionally, N deficiency-induced anthocyanin accumulation disappeared with application of DCMU. These results were further supported by the expression levels of anthocyanin biosynthesis-related genes (*PAL, CHS, CHI, F3H, DFR, UFGT, LDOX*, and *ANS*; **Figure 5**) that will activate anthocyanin biosynthesis, suggesting that N deficiency-induced increase of anthocyanins was Suc-dependent. Sucrose synthase (SS) and sucrose phosphate synthase (SPS) are two important enzymes responsible for sucrose biosynthesis (Lunn and MacRae, 2003). The activity of SS was markedly enhanced by the N deficiency (**Figure 6A**), which provided a positive evidence for our hypothesis while the activity of SPS was not affected (**Figure 6B**).

NR is the first enzyme in the system of transforming inorganic nitrogen into organic nitrogen that would limit the overall nitrogen assimilation in plants (Beevers and Hagemann, 1969). In this study exogenous addition of Suc, no matter under normal condition or N deficiency, reduced the content of soluble protein and the activity of NR (**Figures 6F,H**) suggesting implication of Suc in the process. A significant decrease in glutamine synthase (GS) activity was observed under control (in the presence of N) condition when Suc was added (**Figure 6G**), suggesting inhibition of N metabolic pathway by Suc. Nitrogen and carbon metabolism are tightly linked in almost every biochemical pathway in the plant (Coruzzi and Bush, 2001), and ratio of C/N is generally suggested to be an important parameter for regulation of gene expression (Lea et al., 2007). Results in this study indicate an antagonistic effect between N concentration and Suc content in regulation of anthocyanin biosynthesis. Besides, it was reviewed that ethylene plays a pivotal role in N limitation-induced anthocyanin accumulation by activating PAL activity (Khan et al., 2015). All those hypothesis still requires further investigations.

## Conclusion

N deficiency and high sugar concentration (especially Suc), respectively, or together, have been the focus on the enhancement of anthocyanin accumulation. Though a number of researches reported their separate positive effects on anthocyanin biosynthesis, few of them aimed to investigate the relationship between N deficiency and Suc addition in the regulation of anthocyanin content. In this study, we demonstrated that anthocyanins were accumulated to substantially higher levels under N deficiency in the hypocotyls of radish sprouts. The increased endogenous Suc induced by N limitation and the inhibition effects of DCMU in N deficiency-induced anthocyanin accumulation together implied that when perceived signals from the growth environment with N deficiency, plants synthesized more Suc (most likely through enhancing the activity of SS), which contributed to the increase of anthocyanin content. The detailed mechanism of N deficiency-induced anthocyanin accumulation still remains unclear, while in the present study, we introduce propose and introduce some proof that Suc is an important regulator of this process. Though the relationship between N metabolism and sugar changes in anthocyanin biosynthetic pathway still need further confirmation, the results presented in this research provide the basis to improve our understanding of the regulatory mechanism in anthocyanin biosynthesis that might lead to practical application to production of more nutritious radish sprouts.

## Author Contributions

JC initiated the research. QW designed the research. QW and NS performed the experiments. QW analyzed the data and made all figures. NS wrote the paper, which was revised by JC.

## Conflict of Interest Statement

The authors declare that the research was conducted in the absence of any commercial or financial relationships that could be construed as a potential conflict of interest.
