# Supplementary material for: Increased Sucrose in the Hypocotyls of Radish Sprouts Contributes to Nitrogen Deficiency-Induced Anthocyanin Accumulation
Source: Front Plant Sci. 2016 Dec 26;7:1976. doi: 10.3389/fpls.2016.01976 (PMC5183625; doi:10.3389/fpls.2016.01976)
Supplement: Supplementary file 1 [file Table_1.DOCX]

**Supplementary Table 1.** The nucleotide sequence of primers used in the RT-PCR

| cDNA | Accession number | Primer forward | Primer reverse |
| --- | --- | --- | --- |
| Actin | LOC108863417 | 5’ GCTCAGTCCAAGAGAGGTATTC 3’ | 5’GCTCGTTGTAGAAAGTGTGATG 3’ |
| PAL | UN05484 | 5’GAATTTAACCGCTTCCAACA3’ | 5’AAGCTCAGAGCAGTAAGAAG 3’ |
| CHS | LOC108843267 | 5’CTTGACCGAAGAGTTCTTGA 3’ | 5’GTCTTGTCTAGCATCGAGAG 3’ |
| CHI | LOC108862091 | 5’AACGTTCCCTCCTGGTGCTT 3’ | 5’TTTTCCCGTTTCAGGAATGC 3’ |
| F3H | UN13334 | 5’CAAAATGCCCTCAGCCTGAT 3’ | 5’GCTTGTAAACCACCGACTTGGT 3’ |
| DFR | LOC108826061 | 5’GGATCTGCAGGTTTAACTGA 3’ | 5’TGCGACTATCTGTTTTCTCG 3’ |
| LDOX | LOC108843686 | 5’GTTTGCAGCTTTTCTACGAGG3’ | 5’TGAGCAAAAGTCCGTGGAGG3’ |
| ANS | UN11414 | 5’ACGTAAGCGCTTTGACCTTCA 3’ | 5’ATCGAATCGGGAACGCATT 3’ |
| UFGT | UN33071 | 5’TGTCAGATCGTTTTGGTTCC3’ | 5’GATTCTTCCTCACTTTCTCAC3’ |
|  |  |  |  |
